# A Drug Reaction With Eosinophilia and Systemic Symptoms (DRESS) Syndrome Manifestation in Tubercular Cervical Lymphadenopathy: A Case Report

**DOI:** 10.7759/cureus.67783

**Published:** 2024-08-26

**Authors:** Mahavir Bagrecha, Vinay Balamoni, Siri Vineeth A Ganta

**Affiliations:** 1 Respiratory Medicine, Dr. D. Y. Patil Medical College, Hospital and Research Center, Dr. D. Y. Patil Vidyapeeth, Pune, IND

**Keywords:** anti tubercular treatment, hepatitis, dress syndrome, tuberculosis, adverse reaction, maculopapular rash

## Abstract

A severe adverse reaction linked to a variety of medications, Drug Reaction with Eosinophilia and Systemic Symptoms (DRESS) syndrome is typified by a severe mucocutaneous rash, eosinophilia, fever, lymphadenopathy, and widespread systemic involvement. A 30-year-old female patient presented with fever, facial flushing, and a maculopapular rash that had been persistent for seven days on the upper limb, chest, belly, and lower limbs. She also had a cough with breathlessness for seven days which had progressed over the last seven days and also a rise in temperature in the evening. As the patient was started on anti-tubercular treatment (ATT) based on an interferon-gamma release assay report with no constitutional symptoms, we decided to reconfirm the diagnosis. Ultrasonography of the neck showed cervical lymphadenopathy measuring 21x11 mm in the left cervical region at level IB. The patient was advised for cervical lymph node fine needle aspiration to be sent for cytology and cartridge-based nucleic acid amplification test (CBNAAT) to restart ATT. The patient is being followed up.

## Introduction

The requirement for long-term multidrug regimens, which are linked to a variety of adverse drug reactions (ADRs), complicates tuberculosis chemotherapy. ADRs are also among the most frequent reasons why treatments don't work.

A severe adverse reaction linked to a variety of medications, Drug Reaction with Eosinophilia and Systemic Symptoms (DRESS) syndrome is typified by a severe mucocutaneous rash, eosinophilia, fever, lymphadenopathy, and widespread systemic involvement [1]. ADRs that are frequently linked to anti-tuberculosis drugs (ATDs) include skin responses, arthralgia, influenza-like symptoms, and hepatitis. Severe cutaneous adverse reaction (SCAR) is a subset related to ADR that is immunologically driven and includes toxic epidermal necrolysis (TEN), Stevens-Johnson syndrome (SJS), and DRESS.

ATD-related SCARs are uncommon; however, over the past few years, there has been a steady increase in the number of afflicted cases described in the literature, as well as an increase in DRESS cases connected with ATD [2]. Following this, there is a rash, generalized scaling, facial edema, erythroderma, lymphadenopathy, hematological abnormalities, and damage to the end organs (liver, kidney, heart, lungs, endocrine system, etc.) as well as systemic involvement [3]. Because of its variety of cutaneous manifestations, the "R" in DRESS was altered from rash to reaction/response [4].

Following anti-tubercular treatment (ATT), individuals may occasionally develop severe ADRs, including DRESS syndrome. Awareness of post-ATT DRESS syndrome is essential among healthcare providers to ensure timely recognition and appropriate management. This introduction aims to explore the clinical features, diagnostic challenges, and therapeutic strategies associated with DRESS syndrome in the context of ATT.

A total of 107 cases or 42.13% of the 254 cases that were reviewed and reported in this review, had a connection with ATT drugs. Out of all the agents in this category antiepileptics, allopurinol, sulphonamides, rifampicin, isoniazid, pyrazinamide, and ethambutol were the most frequently reported offending agents [5].

## Case presentation

A 30-year-old female patient presented with complaints of fever, flushing of the face, and maculopapular rash on the upper limb, thorax, abdomen, and lower extremities for seven days. She also had a cough with breathlessness that progressed over the last seven days from grade 1 modified medical research council (MMRC) dyspnea scale to grade 2 MMRC. The fever was insidious, high grade, more so during the evening, and relieved on taking tablet paracetamol 500 mg.

The medical history of the patient revealed that she was started on ATT 15 days previously by an outside physician with the following dosages according to weight band: rifampicin 600 mg once daily, isoniazid 450 mg, pyrazinamide 1000 mg, and ethambutol 800 mg. The patient was diagnosed with cervical tubercular lymphadenopathy by interferon-gamma release assay. The patient also had a history of bronchial asthma since childhood and was managed on a dry powder inhaler formoterol/budesonide 6/200 mcg and used as a reliever and preventer therapy.

**Figure 1:**
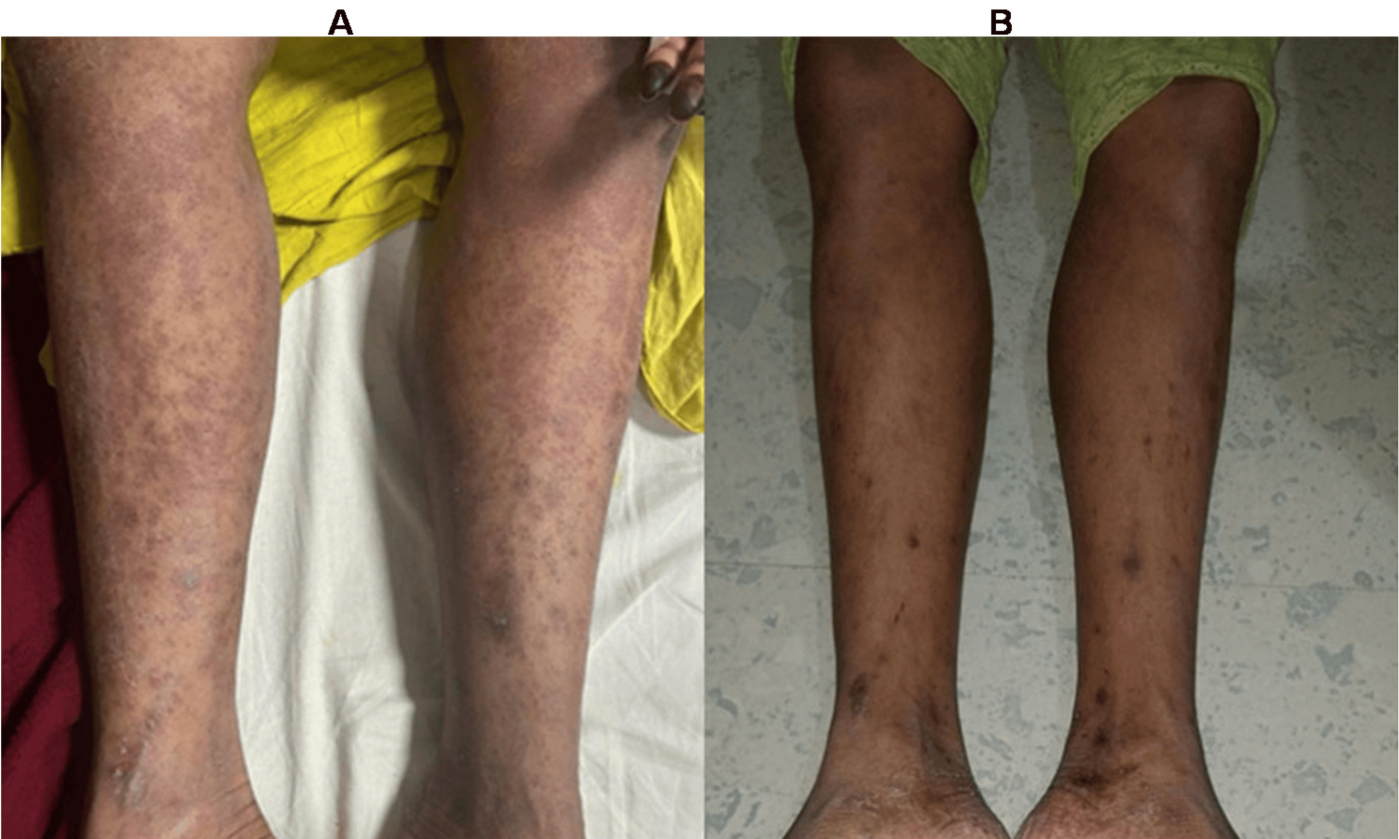
(A) Maculopapular rashes are seen after two weeks of starting ATT drugs; (B) Image of lower limbs after receiving steroids and stoppage of the offending drugs (ATT). ATT: anti-tubercular treatment

On general examination, she had dry skin with erythematous maculopapular lesions over the abdomen, thorax, and upper and lower extremities (Figure 1A). Respiratory system examination revealed bilateral normal vesicular breath sounds. The patient’s vitals were stable. The ultrasonography (USG) of the abdomen revealed findings suggestive of hepato-splenomegaly and mesenteric lymphadenopathy. Blood eosinophil counts, liver enzymes, and creatinine levels were elevated on admission (Table 1).

**Table 1:** Laboratory tests showing elevated liver enzymes, eosinophil count, creatinine

Parameters	Day 1	Day 3	Day 5
Haemoglobin	11.6 g/dl	10.8 g/dl	9.5 g/dl
Total leucocyte count	14200/μL	15100/μL	10600/μL
Platelets (lakhs)	1.28/μL	1.66/μL	2.32/μL
Absolute eosinophil count	1144/μL	1914/μL	623/μL
Eosinophil	13%	22%	7%
Alanine tranaminase	626U/Lt	467U/Lt	232U/Lt
Aspartate transaminase	1307U/Lt	768U/Lt	456U/Lt
Prothrombin time	25.9 seconds	15.7 seconds	12.7 seconds
International normalized ratio	2.25	1.33	1.07
Urea	46 mg/dl	-	23 mg/dl
Creatinine	2.3 mg/dl	-	0.89 mg/dl

Patient was initially diagnosed with ATT-induced hepatitis but after blood tests revealed multiorgan damage with dermatological manifestations, we came to the diagnosis of DRESS syndrome and subsequently the patient was started on oral steroids (tablet prednisolone 0.5 mg/kg) withholding ATT drugs. Blood parameters were monitored for the next five days, which showed a serial decrease in eosinophil count and improvement in liver enzymes, creatinine levels, and skin manifestations (Figure 1B).

As the patient was started on ATT based on interferon-gamma release assay report with no constitutional symptoms, we decided to reconfirm the diagnosis. USG of the neck showed cervical lymphadenopathy with the largest measuring 21x11 mm in the left cervical region at level IB. The patient was advised for cervical lymph node fine needle aspiration to be sent for cytology and cartridge-based nucleic acid amplification test (CBNAAT) before restarting ATT. The patient is being followed up.

## Discussion

DRESS syndrome is a rare but severe hypersensitivity reaction involving multiple organs. It typically presents with fever, rash, and conditions like hepatitis, nephritis, carditis, and pneumonitis. Mortality can reach 10%, particularly with liver involvement, highlighting the need for early diagnosis and treatment [6]. While commonly associated with anticonvulsants and antibiotics, DRESS can also be triggered by ATT such as rifampicin, isoniazid, pyrazinamide, and ethambutol, though this is rare. Diagnosis is clinical and supported by the Registry of Severe Cutaneous Adverse Reactions (RegiSCAR) scoring system, with a score of 5 or more confirming DRESS. Differential diagnoses include SJS and TEN.

Management involves the immediate discontinuation of the offending drug, supportive care, and systemic corticosteroids to control the inflammatory response. Long-term follow-up is crucial to monitor for potential relapses and long-term sequelae. Recognizing the early signs of DRESS and understanding the potential genetic predispositions can aid in prompt diagnosis and effective management, thereby reducing the risk of severe outcomes [7].

ATT-induced DRESS syndrome is typically observed after approximately 15 days of starting treatment. However, in the current case, the patient exhibited symptoms within just seven days. This syndrome has a mortality rate of 8-10%, particularly if the liver is affected. For this reason, prompt diagnosis and treatment are essential. Differentiating DRESS from other skin conditions is crucial. These conditions include viral infections, systemic lupus erythematosus, and scalded skin syndrome, which can occasionally be accompanied by peripheral eosinophilia. Similarly, erythroderma can arise from the aggravation of an underlying skin disorder like psoriasis or atopic dermatitis [8].

A definitive or targeted test to identify DRESS syndrome does not exist. The diagnosis is made based on clinical signs and abnormalities in hematological tests, primarily eosinophilia, as well as abnormalities in the activities of the kidney and liver, if these systems are involved. Moreover, skin biopsy is non-specific [9].

The main challenge in diagnosis is determining the absence of an infectious cause. To rule out a drug hypersensitivity illness, however, a complete history of the patient's medications should be taken in response to skin changes and blood abnormalities. Generally speaking, urticated, maculopapular eruption is the most prevalent skin feature associated with DRESS; nevertheless, reports of vesicles, bullae, pustules, cheilitis, purpura, target lesions, and erythroderma should also be made. One sign of DRESS is facial edema [10].

## Conclusions

DRESS-type delayed hypersensitivity reactions are a significant clinical concern linked to various medications, often emerging in individuals with a genetic predisposition. Due to the potentially severe consequences of DRESS, recognizing its clinical manifestations is crucial for prompt diagnosis and treatment, which helps prevent specific organ damage. Additionally, outpatient follow-up is essential for detecting any post-event sequelae, and treating physicians must diligently monitor these patients.
